# Enrichment and Genomic Characterization of a N_2_O-Reducing Chemolithoautotroph From a Deep-Sea Hydrothermal Vent

**DOI:** 10.3389/fbioe.2018.00184

**Published:** 2018-11-28

**Authors:** Sayaka Mino, Naoki Yoneyama, Satoshi Nakagawa, Ken Takai, Tomoo Sawabe

**Affiliations:** ^1^Laboratory of Microbiology, Faculty of Fisheries Sciences, Hokkaido University, Hakodate, Japan; ^2^Laboratory of Marine Environmental Microbiology, Division of Applied Biosciences, Graduate School of Agriculture, Kyoto University, Kyoto, Japan; ^3^Department of Subsurface Geobiology Analysis and Research (D-SUGAR), Japan Agency for Marine-Earth Science and Technology (JAMSTEC), Yokosuka, Japan

**Keywords:** N_2_O-reducing bacterium, deep-sea hydrothermal field, *Campylobacteria*, *Epsilonproteobacteria*, *nosZ*, nitrous oxide

## Abstract

Nitrous oxide (N_2_O) is a greenhouse gas and also leads to stratospheric ozone depletion. In natural environments, only a single N_2_O sink process is the microbial reduction of N_2_O to N_2_, which is mediated by nitrous oxide reductase (NosZ) encoded by *nosZ* gene. The *nosZ* phylogeny has two distinct clades, clade I and formerly overlooked clade II. In deep-sea hydrothermal environments, several members of the class *Campylobacteria* are shown to harbor clade II *nosZ* gene and perform the complete denitrification of nitrate to N_2_; however, little is known about their ability to grow on exogenous N_2_O as the sole electron acceptor. Here, we obtained an enrichment culture from a deep-sea hydrothermal vent in the Southern Mariana Trough, which showed a respiratory N_2_O reduction with H_2_ as an electron donor. The single amplicon sequence variant (ASV) presenting 90% similarity to *Hydrogenimonas* species within the class *Campylobacteria* was predominant throughout the cultivation period. Metagenomic analyses using a combination of short-read and long-read sequence data succeeded in reconstructing a complete genome of the dominant ASV, which encoded clade II *nosZ* gene. This study represents the first cultivation analysis that shows the occurrence of N_2_O-respiring microorganisms in a deep-sea hydrothermal vent and provides the opportunity to assess their capability to reduce N_2_O emission from the environments.

## Introduction

Nitrous oxide (N_2_O) is a stable greenhouse gas and a major stratospheric ozone layer-depleting substance of the 21th century (Ravishankara et al., 2009). Its 100-years global warming potential is around 300 times higher than CO_2_ (IPPC., 2007). Since atmospheric N_2_O concentration has steadily increased at a rate of 0.2–0.3% per year and this increase is thought be due to anthropogenic emissions (IPCC., 2006), mitigation of N_2_O release is an international challenge in the context of controlling global nitrogen cycle.

Nitrous oxide (N_2_O) can be reduced at the final step of the microbial denitrification pathway, which consists of the sequential reduction of $\text{N}{\text{O}}_{3}^{-}$ to $\text{N}{\text{O}}_{2}^{-}$, NO, N_2_O, and N_2_. N_2_O reductase (NosZ), encoded by *nosZ* gene, is the only known enzyme that converts N_2_O to N_2_ gas. NosZ is phylogenetically classified into two clades; clade I (typical NosZ) and clade II (atypical NosZ). Newly described clade II NosZ (Sanford et al., 2012; Jones et al., 2013) is often identified within non-denitrifying N_2_O-reducing microorganisms that lack other denitrification genes or perform dissimilatory nitrate reduction to ammonium. The abundance of microorganisms possessing the clade II NosZ outnumbers that of microorganisms possessing the clade I NosZ in the diverse environments (Jones et al., 2013, 2014), and clade II organisms have been reported with the higher affinity to N_2_O than clade I organisms (Yoon et al., 2016), suggesting that microorganisms possessing the clade II NosZ play a crucial role in attenuating N_2_O emission in the various natural environments.

Deep-sea hydrothermal vent fields are representative environments where the ecosystem is fueled by chemosynthetic microorganisms that are taxonomically and metabolically diverse (Takai and Nakamura, 2011; Waite et al., 2017; Mino and Nakagawa, 2018). Members of the class *Campylobacteria* are known as one of the predominant bacterial groups there (Muto et al., 2017). Nitrate is a primary electron acceptor for chemosynthetic *Campylobacteria* (Sievert and Vetriani, 2012), and several campylobacterial isolates mediate complete denitrification of $\text{N}{\text{O}}_{3}^{-}$ to N_2_ (Nakagawa et al., 2005; Takai et al., 2006), indicating their ability to reduce N_2_O to N_2_. Clade II *nosZ* gene has been detected in the genomes of several *Campylobacteria* isolated from deep-sea hydrothermal environments (Inagaki et al., 2003, 2004; Nakagawa et al., 2005; Giovannelli et al., 2016). In addition to metabolic and genetic characteristics of the isolates, multiple omics analyses have provided the insights into the *in situ* N_2_O-reducing metabolic potential of *Campylobacteria* (Fortunato and Huber, 2016; Pjevac et al., 2018). The reduction of exogeneous N_2_O, however, has never been characterized for *Campylobacteria* living in deep-sea hydrothermal vents. Here, we, for the first time, report on the direct N_2_O reduction of deep-sea vent chemolithoautotrophs and on its ability of N_2_O consumption.

## Materials and methods

### Sample collection and enrichment of N_2_O-reducing microorganisms

The chimney structure was taken with R/V Yokosuka and DSV Shinkai 6500 from the Baltan chimney at the Urashima site (12°55.3014'N, 143°38.8946'E) in the South Mariana Trough in 2010 during the JAMSTEC cruise YK10-10. After retrieval on board, the sample was anaerobically processed as described previously (Mino et al., 2013). Samples were stored at 4°C until use. Serial dilution cultures were performed using HNN medium. HNN medium contained 0.1% (w/v) NaHCO_3_ per liter of modified MJ synthetic seawater (Sako et al., 1996). Modified MJ synthetic seawater is composed of 25 g NaCl, 4.2 g MgCl_2_ 6H_2_O, 3.4 g MgSO_4_ 7H_2_O, 0.5g KCl, 0.25 g NH_4_Cl, 0.14 g K_2_HPO_4_, 0.7 g CaCl_2_ 2H_2_O, and 10 ml trace mineral solution per liter of distilled water. To prepare HNN medium, concentrated solution of NaHCO_3_ was added before gas purging of 100% N_2_O. The tubes were then tightly sealed with butyl rubber stopper, autoclaved, and pressurized the headspace to 300 kPa with 80% H_2_ + 20% CO_2_. H_2_ is a sole energy source of the medium. The enrichment was performed with HNN medium under 33°C. The N_2_ production under N_2_O-reducing condition was confirmed using a Shimazu GC-2014 gas chromatograph (Shimadzu, Kyoto, Japan).

### Cultivation experiments for the community analysis

The microbial community was precultured in HNN medium for 24 h at 38°C. Then, 2 mL of preculture was inoculated to 50 mL glass vials each containing 20 mL medium (33% N_2_O + 54% H_2_ + 13% CO_2_, 300 kPa). 5 vials were cultivated at once at 38°C, with mixing using a magnetic stirrer (MS-51M; AS ONE, Osaka, Japan) at 850 rpm. The concentration of headspace N_2_O of each cultivation vial at each time point (*t* = 0, 12, 18, 24, 36, 42, and 48 h) was measured using a gas chromatograph (GC-2014; Shimadzu, Kyoto, Japan) with the SHINCARBON ST 50/80 (2 m × 3 mmϕ) column (Shinwa Chemical Industries, Kyoto, Japan). The cultivation was continued until decrease of N_2_O concentration reaches a plateau. At each time point (*t* = 12, 18, 24, 36, and 48 h), one of the replicate vials was used and 20 mL of culture medium was transferred into 50 ml plastic centrifugation tubes. After centrifugation at 10,000 × g for 20 min, the supernatant was removed and cell pellet was stored at −30°C until DNA extraction. Cultivation experiment was carried out in triplicate.

### DNA extraction

DNA extraction from the pelleted cell culture samples were performed with NucleoSpin Soil DNA kit (Macherey-Nagel, Düren, Germany) according to the protocol provided by the manufacturer. The DNA samples were stored at −30°C until sequencing analysis. A total of 15 DNA samples was used for 16S rRNA gene-based microbial community analysis. Four DNA samples from two time points (*t* = 12 and 36 h, *n* = 2) were also used for metagenomic analysis.

### Illumina 16S rRNA gene region amplification and sequencing

Each amplicon library was constructed by amplifying V1-V2 paired-end libraries using 27Fmod (5′-AGRGTTTGATYMTGGCTCAG-3′) and 338R (5′-TGCTGCCTCCCGTAGGAGT-3') according to Nextera library preparation methods. Equal amounts of each PCR amplicons were pooled, followed by amplification and 2 × 300 bp paired-end sequencing on the MiSeq platform according to the manufacturer's instructions.

### Data analysis of 16S rRNA gene amplicons

A total of 519,290 raw reads across 15 samples, with an average 34,619 reads per sample, was further processed by QIIME 2 software package (version 2018.4). Quality control was performed using DADA2 (Callahan et al., 2016) by removing chimeras and residual PhiX reads, and low-quality regions of sequences. Forward and reverse reads were, respectively, truncated to 230 and 209 bp for DADA2 analysis based on the average quality scores determined. After quality filtering, the dataset contained 421,406 reads, with an average of 28,093 sequences per samples. Dereplication was then performed by DADA2, which combines identical reads into amplicon sequence variants (ASVs), which is analog to the traditional Operational Taxonomic Unit (OTU). Representative sequences were classified into taxonomic groups using the SILVA 132 database. Mitochondrial and chloroplast sequences were then removed from the feature table, and bacterial and archaeal sequences were used further analysis. We used BLASTN search against the online nucleotide collection (nr/nt) database on NCBI to find the closest GenBank neighbor for these representative sequences.

### Metagenome analyses by illumina and nanopore sequencing

For two replicates from two time points (*t* = 12 and 36 h), paired-end libraries for metagenome sequencing were generated using Nextera library preparation methods. Genome sequencing was then performed on an Illumina MiSeq platform (2 × 300 bp paired-end). Read data from Illumina sequencing were trimmed with Platanus_trim (Kajitani et al., 2014).

Nanopore sequencing was conducted with a DNA sample from a time point 36 h (*n* = 1). Sequencing library preparation was carried out using the Rapid Barcoding Sequence kit (SQK-RBK001) (Oxford Nanopore Technologies, Oxford, UK) according to the standard protocol provided by the manufacturer. The constructed library was loaded into the FlowCell (FLO-MIN106) on a MinION device and performed 24 h sequencing run with MinKNOW1.7.14 software. The fast5 read file was basecalled with ONT Albacore command line tool (v2.0.2). The adapter sequences were trimmed from the fastq read file using Porechop v0.2.1 (https://github.com/rrwick/Porechop).

### Construction of metagenome assembled genome (MAG)

For assembly, Canu version 1.6 (Koren et al., 2017) was run using Nanopore reads with following options: genome size 2.1 Mb, and correctedErrorRate = 0.120. The Illumina short reads were mapped to the Canu's assembly by BWA version 0.7.12 (Li, 2013). Pilon version 1.22 (Walker et al., 2014) was run on the alignments to polish the assembly sequences. Circlator version 1.5.1 (Hunt et al., 2015) program was used for circularizing the genome assembly. Two contigs were again polished by Pilon using Illumina reads. Metagenome assembled genome (MAG) sequences were assessed using CheckM v1.0.8 (Parks et al., 2015) for the completion estimate, and then larger MAG was annotated with the Rapid Annotation using Subsystem Technology (RAST) server v2.0 (Aziz et al., 2008). rRNA genes were predicted using RNAmmer (Lagesen et al., 2007). 16S rRNA gene sequences of the MAG were compared to a representative sequence of a dominant ASV obtained by 16S rRNA amplicon analysis. MAG was functionally annotated using KEGG database with the BlastKOALA pipeline (Kanehisa et al., 2016) and KO numbers were mapped using KEGG mapper to compare the metabolic pathway with a closely related species.

### Taxonomic analysis of MAG

Taxonomic placement of the MAG was assessed based on both 16S rRNA and genomic approaches. 16S rRNA gene sequence of the MAG was aligned to closely related species using SILVA Incremental Aligner v1.2.11 (Pruesse et al., 2012). A phylogenetic tree was constructed by Maximum Likelihood (ML) method using MEGA 7.0.26 package (Kumar et al., 2016) with GTR+I+G substitution model. Phylogenomic tree construction was performed using PhyloPhlAn (Segata et al., 2013), which extracts up to 400 conserved proteins coded in the genomes of closely related species and generates multiple alignments of each protein. Multiple alignments of each universal protein were then concatenated into a single amino acid sequence. The optimal model for phylogenomic analysis was determined by Modelgenerator (Keane et al., 2006), and then ML tree was constructed using RAxML version 8.2.11 (Stamatakis, 2014) with BLOSUM62+G+F model. Average nucleotide identity (ANI), average amino acid identity (AAI) and *in-silico* DNA-DNA hybridization (DDH) values were calculated by ANI calculator (http://enve-omics.ce.gatech.edu/ani/index), AAI calculator (http://enve-omics.ce.gatech.edu/aai/) and Genome-to-Genome Distance Calculator (GGDC 2.1) (Meier-Kolthoff et al., 2013), respectively.

### Comparison of *nos* clusters and *nosZ* gene sequences within related *campylobacteria*

The genomes of chemosynthetic *Campylobacteria* were used to reconstruct the *nos* gene clusters. Sequences of functionally characterized NosZ protein and its accessory genes from *Wolinella succinogenes* were used to search the genes adjacent to inferred *nosZ* on *Hydrogenimonas* sp. MAG. Multiple amino acid sequence alignment was generated from representatives of clade I and clade II *nosZ* sequences using MUSCLE version 3.8.31 (Edgar, 2004). ML tree based on *nosZ* amino acid sequences was constructed by MEGA with WAG+G model based upon 500 bootstrapped replicates. Trees were visualized using FigTree v1.4.2 (http://tree.bio.ed.ac.uk/software/figtree/).

### Growth characteristics

In attempt to evaluate the nitrate-respiring ability of the enrichment, each of the potential electron donors, such as H_2_, elemental sulfur (1%, w/v), or thiosulfate (0.1%, w/v) was tested with nitrate (0.1%, w/v) as an electron acceptor under 40°C. The ability to use H_2_ was examined with 80% H_2_ + 20% CO_2_ (300 kPa) in the headspace. For testing growth on elemental sulfur and thiosulfate as an electron donor, 80% N_2_ + 20 % CO_2_ was used as the gas phase (300 kPa).

## Results and discussion

### N_2_O consumption and community structure of the enrichment culture

**Figure 1:**
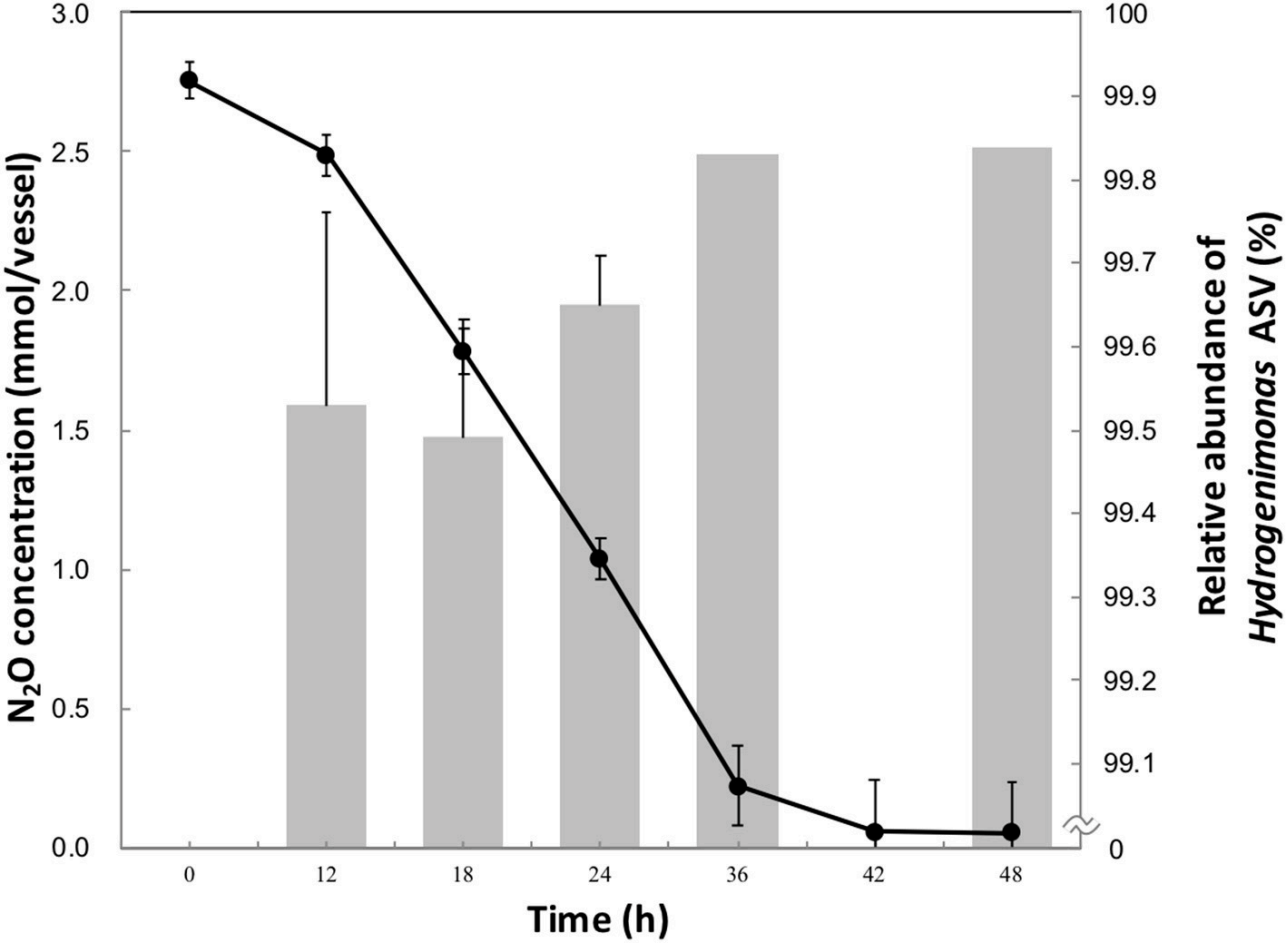
N_2_O consumption (•) and relative abundance of *Hydrogenimonas* ASV in the enrichment culture (bar chart) during the 48 h-cultivation. The data points and error bars represent the means and standard errors, respectively.

Approximately 90% of initial headspace N_2_O was consumed after 42 h of cultivation (Figure 1). The maximum rate of N_2_O consumption was 3.3 μmol h^−1^ per ml of culture, higher than other clade I and II reducers (Sanford et al., 2012; Yoon et al., 2016), though a higher initial N_2_O concentration in this study was provided than in the previous studies. The aqueous-phase N_2_O concentration was calculated from the Ostwald coefficient (0.4190) at 1 atm partial pressure and 40°C (Wilhelm et al., 1977). 2.75 mmol of vessel N_2_O in 20 ml of medium corresponded to an initial aqueous-phase N_2_O concentration 56.5 mM. This concentration is more than million-fold higher than the N_2_O level observed in deep-sea hydrothermal environments, where N_2_O is mainly supplied by microbial nitrification and denitrification processes (Lilley et al., 1982; Kawagucci et al., 2010).

To identify the N_2_O-reducing organisms in the enrichment culture, we analyzed the time-series community structure during the cultivation under the HNN medium. Dereplicate 421,406 Illumina reads resulted in 11 bacterial ASVs. The genus *Hydrogenimonas* consisting of a single ASV was the most predominant bacterial group through the cultivation period and its relative abundance was above 98% (Figure 1 and Figure S1). In order to further identify this dominant ASV, a representative sequence was extracted from the sequence data and analyzed by BLAST search. The best match represented *Hydrogenimonas thermophila* EP1-55-1%^T^ (= JCM 11971^T^ = ATCC BAA-737^T^) with 90% identity, suggesting the host organism of the ASV would be a new species of *Hydrogenimonas* or even a new genus of the class *Campylobacteria*. Strain EP1-55-1%, isolated from a deep-sea hydrothermal vent field in the Indian Ocean, is able to utilize nitrate as an electron acceptor and shows the optimum growth at 55°C (Takai et al., 2004). These characteristics are different from the N_2_O-reducing enrichment culture in this study, which shows an optimum growth at 40°C and no growth under nitrate-respiring condition. The genus *Thalassospira* consisting of a single ASV accounted for about 0.7% of reads at 12 h, and its abundance gradually decreased to 0.1% at 36 h of cultivation. The representative sequence of the ASV assigned to *Thalassospira* showed 100% similarity to *T. xianhensis* P-4^T^ (= CGMCC 1.6849^T^ = JCM 14850^T^) isolated from an oil-polluted saline soil (Zhao et al., 2010). *T. xianhensis* engages denitrification, possibly having N_2_O-reducing ability, but it cannot grow under autotrophic condition. Along with the increase of relative abundance of *Hydrogenimonas*, headspace N_2_O decreased and reached 0.05 mmol/vessel at 48 h, showing that members belonging to *Hydrogenimonas* significantly contribute to N_2_O consumption of the enrichment culture.

### Reconstruction of the genome, taxonomy, and denitrification pathway of a novel N_2_O-reducing campylobacterium strain BAL40

In order to understand the genomic characteristics of most dominant ASV species, we employed the MAG reconstruction strategy, because cells formed pellicle-like structures that was unable to be purified to a single pure strain by the dilution-to-extinction technique. High-quality 14,951,908 reads (4.6 Gb) and 236,546 reads (1.1 Gb) of metagenomic data were obtained by Illumina and Nanopore sequencing, respectively, and assembled into two contigs. After circularization, a larger contig resulted in a complete circular, which is 2,193,435 bp in length with an average G + C content of 50.2% and >99% genome completeness (Figure S2). In total, 2,185 coding sequence (CDS) regions and 54 RNAs were annotated. Three copies of 16S rRNA gene were annotated by RNAmmer, and its V1-V2 regions were identical with each other and with the represent sequence of the dominant ASV. With full length of 16S rRNA gene sequences as a query in BLAST search, the highest similarity was estimated with *H. thermophila* EP1-55-1%^T^ (94%). The size of the other contig is 49,484 bp with 0% completeness level estimated by CheckM.

**Figure 2:**
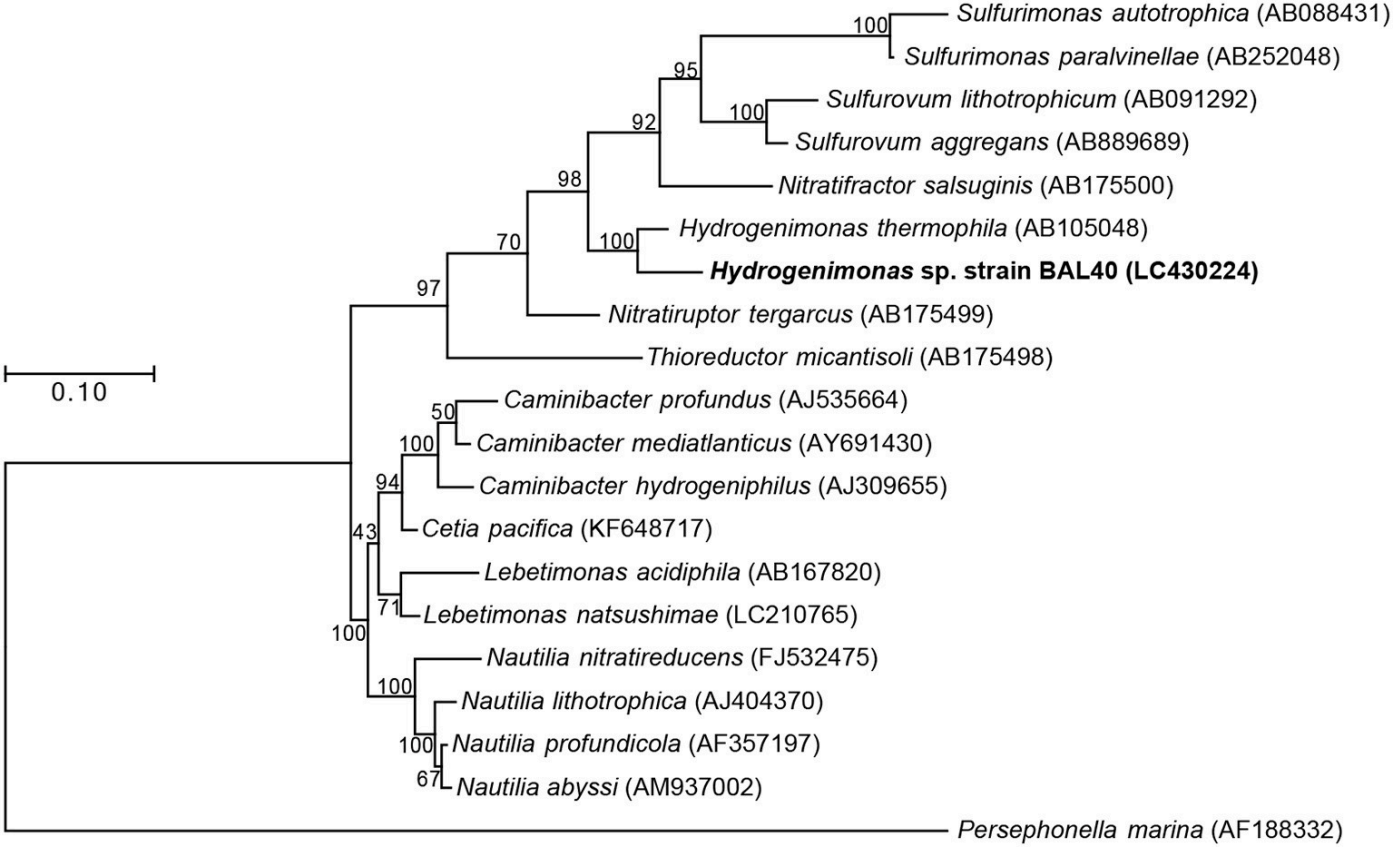
ML Phylogenetic tree based on 1,196 nucleotide position of 16S rRNA gene sequences. Bootstrap values based on 500 resampling replicates are shown as percentages at branch nodes.

**Figure 3:**
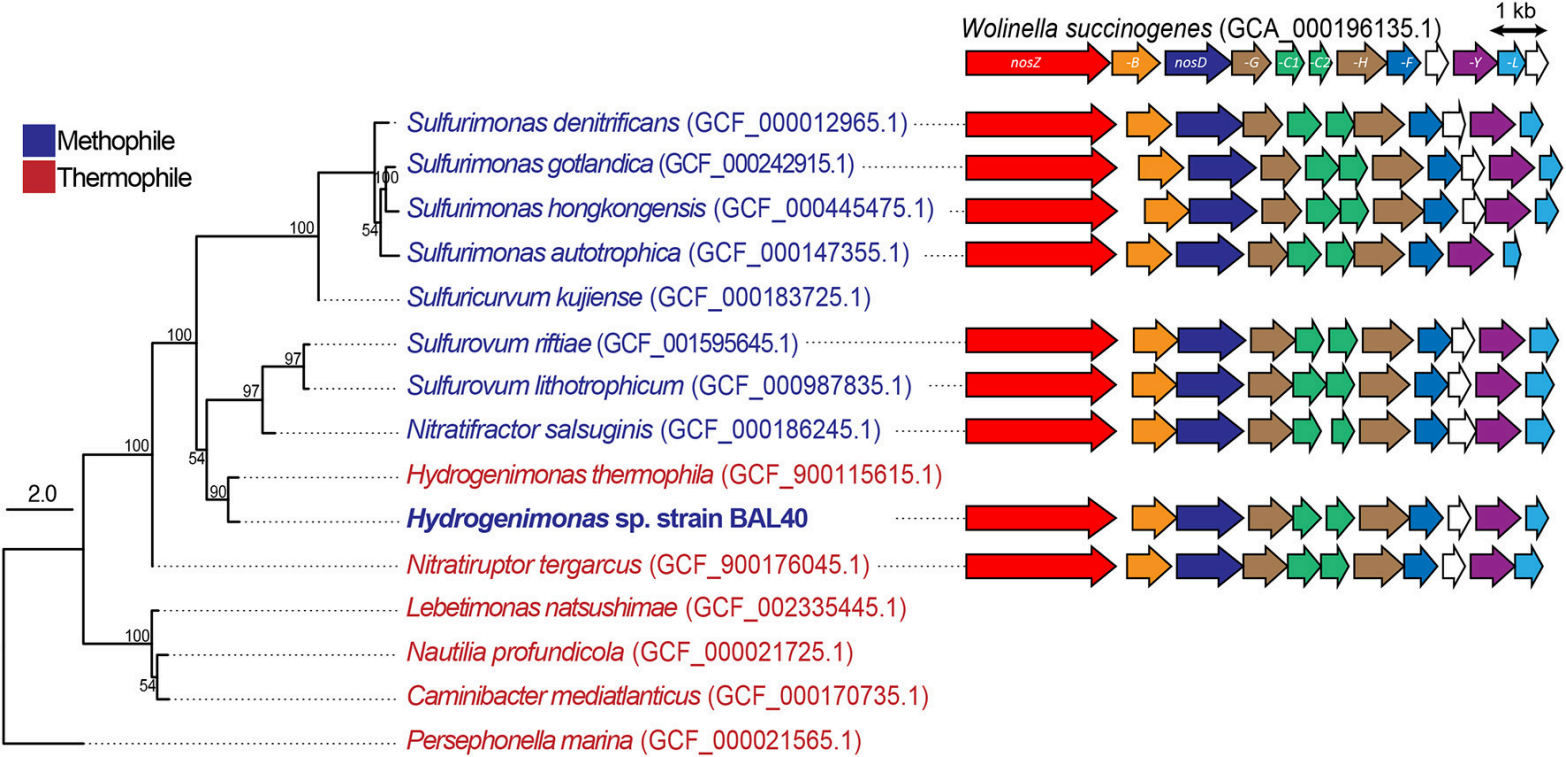
ML tree based on genome sequences and schematic comparison of the *nos* gene clusters in different *Campylobacteria*. Bootstrap values based on 500 resampling replicates are shown as percentages at branch nodes. Clade II *nosZ* and its accessory genes (BDFGHLY) are labeled and colored according to homology across the different species.

Both 16S rRNA gene-based and genome-based phylogenetic trees revealed that the MAG was most closely related to *H. thermophila* (Figures 2,3). *In-silico* DDH and ANI values between the strain BAL40 MAG and *H. thermophila* genome sequences were 17.4% and 74.4%, respectively, well below species cutoff (Richter and Rosselló-Móra, 2009). AAI value with *H. thermophila* was 71.4%, which is higher than the genus criterion level (Konstantinidis and Tiedje, 2005; Konstantinidis et al., 2017). Taken together, the strain BAL40 MAG described here represents a novel species within the genus *Hydrogenimonas*.

Reconstruction of denitrification pathway using KEGG database revealed that the MAG possessed whole set of denitrification genes (*nap, nir, nor*, and *nos*) (Figure S3), suggesting the ability to utilize nitrate as an electron acceptor. However, the enrichment culture did not show any growth under the nitrate-respiring condition in this study. This discrepancy between genetic and physiological characteristics implies the existence of heretofore overlooked N_2_O-reducing microorganisms, which are genetically denitrifiers *sensu stricto* but are physiologically non-denitrifiers. However, the influence of *in situ* physicochemical conditions on their denitrification metabolisms could not be examined here. Considering our results, we speculate that together diverse microbial species drive different steps to the full denitrification pathway of nitrate to N_2_ in deep-sea hydrothermal environments. It should be noted that because most environmental studies investigating abundance, diversity and function of denitrifiers have been performed based solely on the PCR-based marker gene analysis (Braker and Tiedje, 2003; Henry et al., 2006; Smith et al., 2007; Sanford et al., 2012; Jones et al., 2013), it is virtually impossible to identify which organism is actually responsible for specific steps of the denitrification process. Further culture-dependent studies could demonstrate the actual function of both denitrifying and non-denitrifying microorganisms. In addition, cultivation under N_2_O-respiring condition might decrease the number of yet-to-be cultured microorganisms and unveil the N_2_O mitigation potential hidden in deep-sea hydrothermal environments.

### Reconstruction of the *nos* gene cluster and comparison of *nosZ* sequences in *campylobacteria*

We reconstructed *nos* gene clusters from the genomes of MAG and related *Campylobacteria* including *Wolinella succinogenes*, which has been used as a campylobacterial model organism (Figure 3). Similar to *W. succinogenes, Hydrogenimonas* sp. MAG possessed a clade II *nos* gene cluster that contains *nosZ, -B, -D, -G, -H, -C1, -C2, -H, -Y, -L* genes, and the presence and organization of these genes were well-conserved in the related *Campylobacteria* members, as previously described (Torres et al., 2016). A phylogenetic tree based on amino acid sequences of *nosZ* gene indicated that the sequence of *Hydrogenimonas* sp. MAG is closely related to *Nitratifractor salsuginis* E9I37-1^T^, which is known as a nitrate-reducing mesophile (Nakagawa et al., 2005) (Figure S4). Multiple alignment of NosZ amino acid sequences that contained both clade I and clade II sequences showed that NosZ of *Campylobacteria* including *Hydrogenimonas* sp. MAG conserved ligands of two cooper sites; Cu_A_ electron transfer center and Cu_Z_ catalytic center (Zumft and Kroneck, 2007) (Figure S5). It is therefore deduced that N_2_O-respiring metabolism supports more diverse *Campylobacteria* in deep-sea hydrothermal environments.

### N_2_O emission and sink in deep-sea hydrothermal environments

A variety of microorganisms contributing to the denitrification process have been reported in a wide range of environments such as sediments, paddy soils, hydrothermal vents, and geothermal streams (Shao et al., 2010; Masuda et al., 2017; Kato et al., 2018). N_2_O emissions by denitrification are the net result of the balance between its production and reduction to N_2_. In deep-sea hydrothermal environments, diverse microorganisms including *Campylobacteria* involved in denitrification processes (Bourbonnais et al., 2012; Bowles et al., 2012; Fortunato and Huber, 2016; Pjevac et al., 2018). The occurrence of microorganisms with the high ability to reduce exogenous N_2_O could explain the previous physicochemical results that *in situ* N_2_O level is quite low (Lilley et al., 1982; Kawagucci et al., 2010), and its flux to the deep waters is negligible (Bange and Andreae, 1999). Our findings imply the capacity of deep-sea hydrothermal environments to act as a sink for N_2_O is greater than its capacity to emit N_2_O.

## Conclusion

We conducted, to the best of our knowledge, the first culture-dependent study of deep-sea hydrothermal vent N_2_O-reducing microorganisms. Evaluation of N_2_O consumption ability and genome reconstruction from metagenomic data demonstrated that the notable N_2_O consumption rate of the novel member within genus *Hydrogenimonas*. Our findings will open door for new ideas to implement biotechnological applications of NosZ from thermophiles, such as nitrogen removal from wastewater. Future studies, such as comparative cultivation analysis of N_2_O-reducing *Campylobacteria*, transcriptional analysis of *nos* genes, and measurement of N_2_O consumption kinetics, may provide the insights into the mechanisms allowing to their high N_2_O consumption ability.

## Data availability

This project has been deposited at DDBJ/EMBL/GenBank under the BioProject PRJDB7296. Sequences for MAG and 16S rRNA gene of *Hydrogenimonas* sp. strain BAL40 are available with DDBJ/EMBL/GenBank AP019005 and LC430224, respectively.

## Author contributions

SM, SN, and TS designed research. SM, NY performed the experiments and analyzed the data. SN and KT contributed the sample collection. SM and TS contributed reagents, materials, and analysis tools. SM wrote the paper with inputs from SN, KT, and TS.

### Conflict of interest statement

The authors declare that the research was conducted in the absence of any commercial or financial relationships that could be construed as a potential conflict of interest.
