# Supplementary material for: Enrichment and Genomic Characterization of a N2O-Reducing Chemolithoautotroph From a Deep-Sea Hydrothermal Vent
Source: Front Bioeng Biotechnol. 2018 Nov 28;6:184. doi: 10.3389/fbioe.2018.00184 (PMC6279868; doi:10.3389/fbioe.2018.00184)
Supplement: Supplementary file 1 [file Data_Sheet_1.PDF]

Supplementary Figures

Enrichment and genomic characterization of a N_2O-reducing chemolithoautotroph from a deep-sea hydrothermal vent

Sayaka Mino, Naoki Yoneyama, Satoshi Nakagawa, Ken Takai, and Tomoo Sawabe

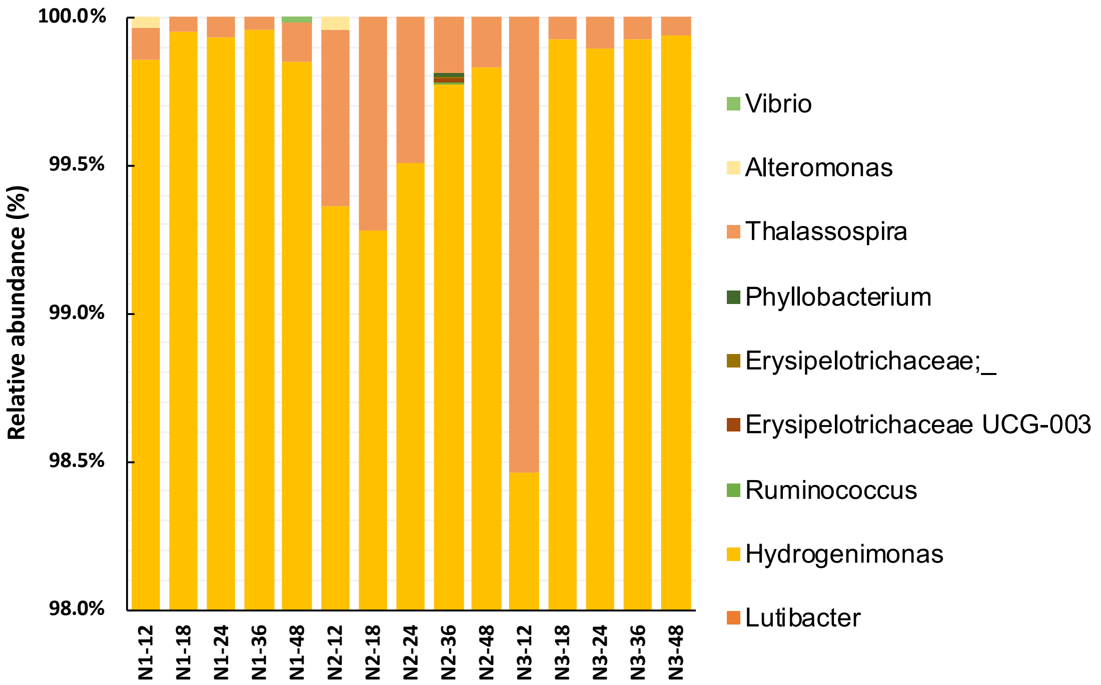

Figure S1. Relative ASV abundance of different genus in the N_2O-reducing community after 12 h to 48 h cultivation.

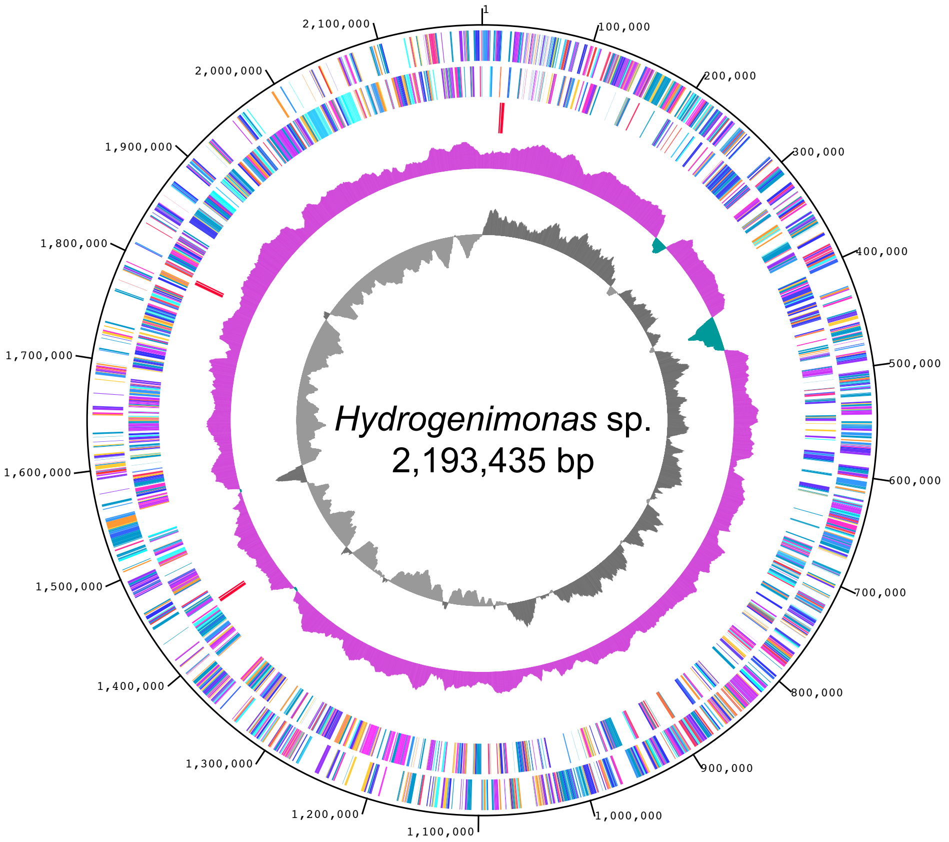

Figure S2. Graphical circular map of the *Hydrogenimonas* sp. MAG. From outside to the center: genes on forward strand (color by COG categories), genes on reverse strand (color by COG categories), rRNA genes, G + C content, GC skew.

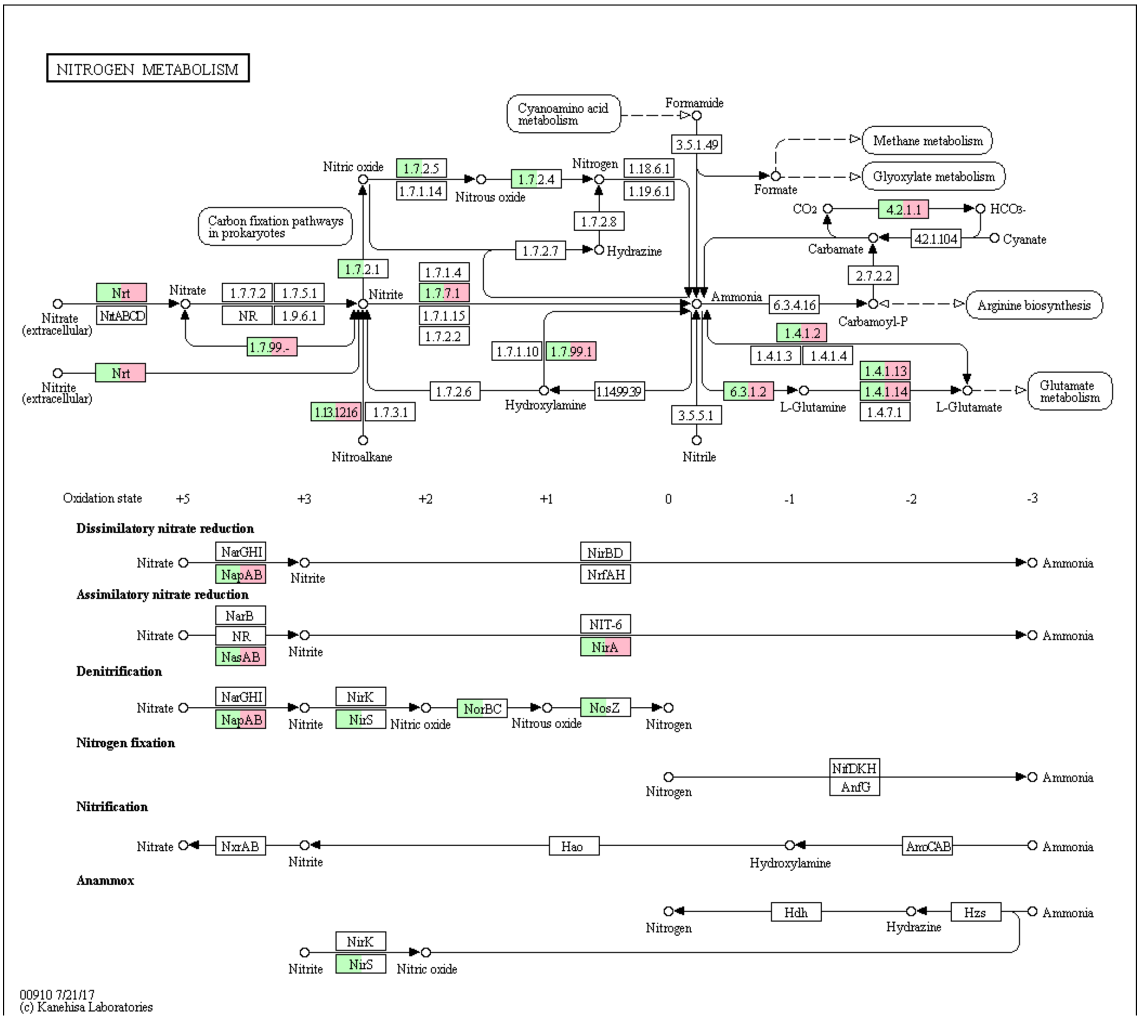

Figure S3. Nitrogen metabolism pathway of *Hydrogenimonas* as defined in KEGG pathway database. Highlighted boxes in green and red are the enzymes found in *Hydrogenimonas* sp. strain BAL40 and *H. thermophila*, respectively.

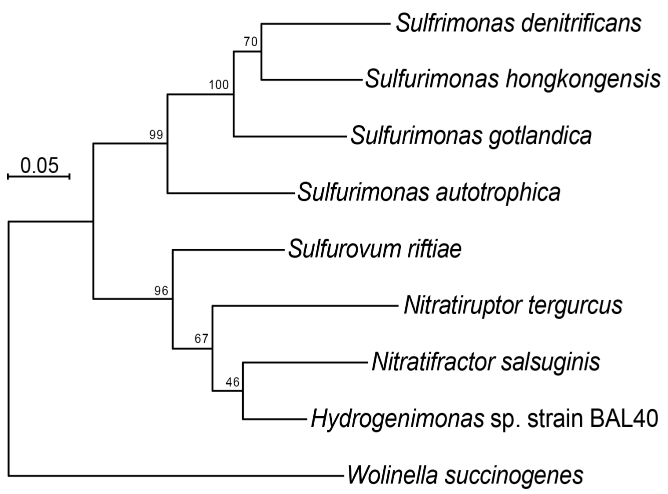

Figure S4. ML phylogenetic tree based on 862 amino acid position of *nosZ* sequences of relative *Campylobacteria*. Bootstrap values based on 500 resampling replicates are shown as percentages at branch nodes.

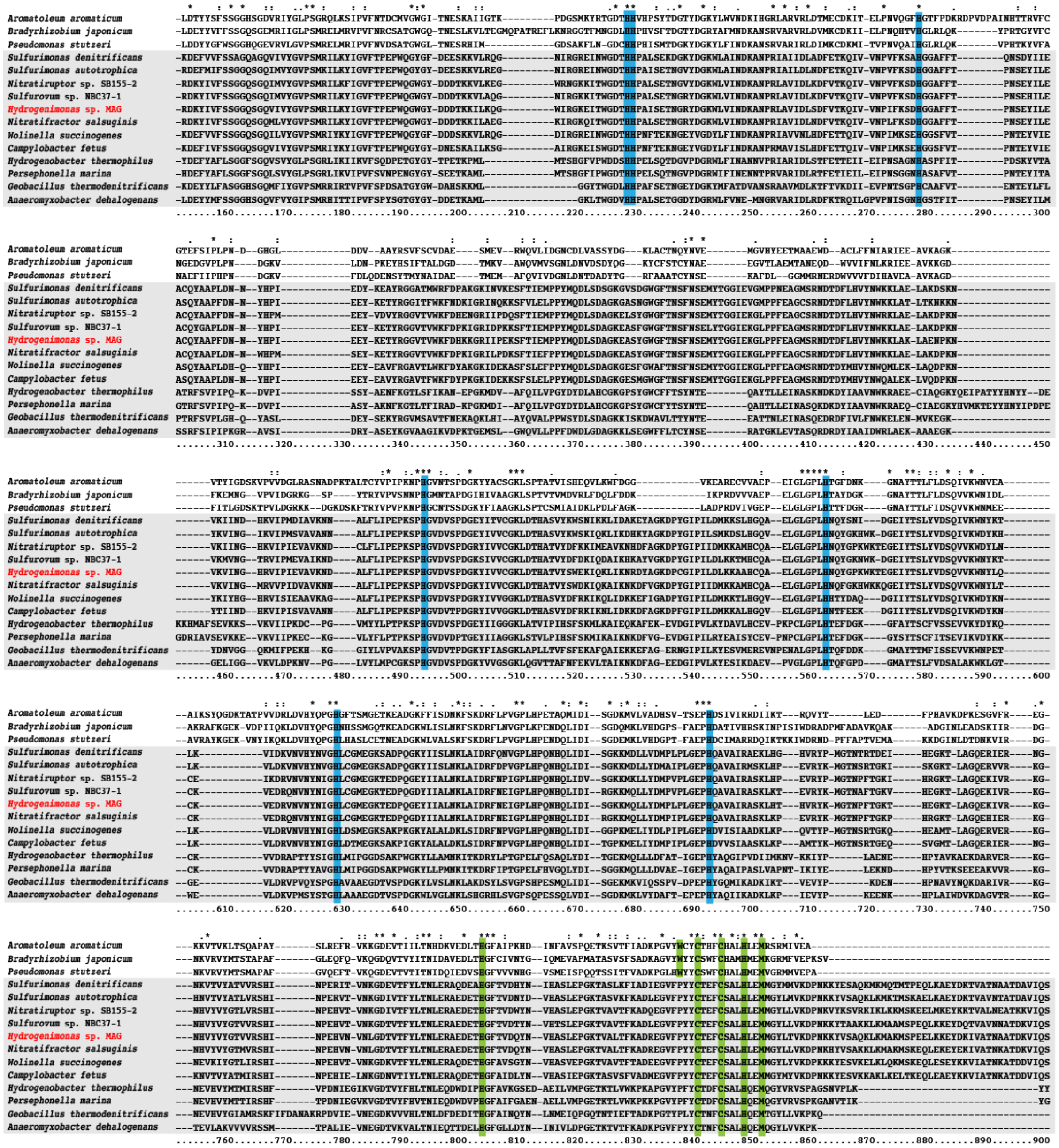

Figure S5. Alignment of NosZ primary structure of *Hydrogenimonas* sp. strain BAL40 and other clade I and clade II represents. Clade II NosZ sequences are highlighted in gray. Conserved seven histidine ligands of the Cu_Z center are highlighted in blue. Conserved two cysteine and other ligands of the Cu_A center are highlighted in green.
